# Probing the Carbonyl Functionality of a Petroleum Resin and Asphaltene through Oximation and Schiff Base Formation in Conjunction with N-15 NMR

**DOI:** 10.1371/journal.pone.0142452

**Published:** 2015-11-10

**Authors:** Kevin A. Thorn, Larry G. Cox

**Affiliations:** U.S. Geological Survey, Denver Federal Center, MS 408, Denver, Colorado, 80225–0046, United States of America; University of Michigan, UNITED STATES

## Abstract

Despite recent advances in spectroscopic techniques, there is uncertainty regarding the nature of the carbonyl groups in the asphaltene and resin fractions of crude oil, information necessary for an understanding of the physical properties and environmental fate of these materials. Carbonyl and hydroxyl group functionalities are not observed in natural abundance ^13^C nuclear magnetic resonance (NMR) spectra of asphaltenes and resins and therefore require spin labeling techniques for detection. In this study, the carbonyl functionalities of the resin and asphaltene fractions from a light aliphatic crude oil that is the source of groundwater contamination at the long term USGS study site near Bemidji, Minnesota, have been examined through reaction with ^15^N-labeled hydroxylamine and aniline in conjunction with analysis by solid and liquid state ^15^N NMR. Ketone groups were revealed through ^15^N NMR detection of their oxime and Schiff base derivatives, and esters through their hydroxamic acid derivatives. Anilinohydroquinone adducts provided evidence for quinones. Some possible configurations of the ketone groups in the resin and asphaltene fractions can be inferred from a consideration of the likely reactions that lead to heterocyclic condensation products with aniline and to the Beckmann reaction products from the initially formed oximes. These include aromatic ketones and ketones adjacent to quaternary carbon centers, β-hydroxyketones, β-diketones, and β-ketoesters. In a solid state cross polarization/magic angle spinning (CP/MAS) ^15^N NMR spectrum recorded on the underivatized asphaltene as a control, carbazole and pyrrole-like nitrogens were the major naturally abundant nitrogens detected.

## Introduction

Determination of the molecular structures of the asphaltene fraction of crude oil has been a long term goal in petroleum geochemistry and engineering [1–4]. Knowledge of structures is a prerequisite to understanding the chemical properties of asphaltenes and their role in imparting viscosity to crude oil, fouling pipes during petroleum refining, and impeding extraction of oil by the clogging of rocks in oil field reservoirs. There has also been an increasing interest in the asphaltene and resin fractions of crude oil in the context of environmental pollution from oil spills. As researchers attempt to gain a more complete understanding of the fate of spilled crude oil, the pathways and extent to which asphaltenes and resins undergo microbial and photochemical degradation need to be elucidated [5–16]. This is especially true in view of the developing worldwide dependence on heavy crudes as sources of light crude oil become depleted. In North America, with the expected increase in transportation throughout the continent of diluted bitumen from the Alberta Tar Sands for example, spills of heavy crudes such as the Enbridge incident on the Kalamazoo River [17] may become more common place. In marine spills, asphaltenes have long been recognized as constituents of tarballs. EPR signals characteristic of asphaltenes were detected in tarballs collected from the Deep Water Horizon spill for example [18].

With its ability to provide molecular formulas through ultrahigh resolution, Fourier-transform ion cyclotron resonance mass spectrometry (FTICR-MS) has contributed significant information on the total numbers of individual structures, carbon number and molecular weight ranges, and heteroatom compound classes in asphaltenes [3, 19–21]. Asphaltenes may contain up to 10,000 individual structures, compared to the largest number of total structures reported for a whole crude oil thusfar of approximately 85,000 [22]. FTICR-MS and other techniques have been consistent in revealing a molecular weight range for asphaltenes from approximately 500 to 2000 Daltons that translates into an approximate carbon number range from C_35_ to C_100_. From 50 to 80% of molecular formulas of asphaltenes provided by FTICR-MS contain at least one heteroatom (O,S,N) and approximately half contain two or more heteroatoms. FTICR-MS analyses have revealed that asphaltenes share the same carbon number ranges as their corresponding maltenes but that the asphaltene molecules contain greater aromaticities, supporting the Boduszynski continuum model [20]. NMR analyses have provided support for the “island” model of asphaltenes, in which the asphaltene molecules are viewed as consisting of a large PAH core with aliphatic side chains attached [23,24]. It has been argued, however, that evidence in support of the archipelago model (smaller PAH cores connected through aliphatic bridges) cannot be entirely discounted [21]. Appearing to support this line of reasoning, atomic force microscopy (AFM) analyses indicated a predominance of island structures but also confirmed the presence of archipelago structures in coal and petroleum asphaltenes [25]. A recent FTICR-MS study of the resin fractions from two vacuum gas oils indicated a carbon number range of C_13_ to C_24_ with molecular weight distributions centered at 350 Da and 450 Da. Structures with molecular formulas containing only one oxygen, nitrogen or sulfur were the most abundant heteroatom classes [26]. Alteration of acidic and neutral polar NSO compounds in general through subsurface anaerobic biodegradation in reservoirs has been revealed by FTICR-MS [27].

Heteroatom functional groups detected in heavy petroleum fractions have been compiled in several reviews [1, 4, 28]. From a number of analytical techniques, oxygen has been shown to occur in the form of carboxylic acids, phenolic hydroxyls, ketones, esters, cyclic and acyclic ethers, amides and sulfoxides. Quinones have been inferred from electrochemical studies [29]. Relatively few studies have reported concentrations of these functional groups in asphaltenes and resins [30].

Here we examine the carbonyl functionality of a petroleum resin and asphaltene (pentane precipitate) through reaction with ^15^N-labeled hydroxylamine and aniline in conjunction with analysis by ^15^N NMR, an approach previously applied to humic and fulvic acids [31,32], and a technique that complements established ^13^C NMR spin labelling procedures for determination of acidic oxygen, carbon, nitrogen and sulfur groups in the heavy petroleum fractions. In IR (Infrared) spectroscopy, the carbonyl stretches of carboxylic acids, ketones, quinones and esters are not completely resolved from one another. Nitrogen-15 NMR detection of ketones via their oxime derivatives and quinones via their anilinohydroquinone adducts overcomes this problem. The ^15^N NMR technique also has potential as a tool to follow the oxidation of asphaltenes and resins via biodegradation and exposure to solar radiation. The oximation method was used for example to document the preferential loss of the quinone/hydroquinone functionality over ketones in aquatic natural organic matter subjected to UV irradiation [33,34]. The analytical challenge in this study is the low concentration of carbonyl groups in the asphaltene and resin fractions, an order of magnitude less than in humic substances.

The asphaltene and resin examined here were isolated from a light aliphatic crude oil that contaminated groundwater near Bemidji, Minnesota, as a result of a pipeline break in August of 1979 [35]. Subsurface biodegradation of the crude oil at the Bemidji site has been the subject of an ongoing multidisciplinary research effort since 1983 [36,37]. One of the most notable features documented at the site has been the development, downgradient from the oil body floating atop the groundwater, of a plume of dissolved organic carbon (DOC) that corresponds to the partial oxidation products of the crude oil constituents in the form of nonvolatile organic acids. The sulfur contents of the nonvolatile organic acids indicated that they derived in part from the sulfur-containing constituents of the crude oil, which could possibly include the asphaltene and resin fractions [35]. The NMR analyses of the asphaltene and resin are part of an ongoing effort to characterize the Bemidji crude oil further, including FTICR-MS studies, in the larger context of relating the partial degradation products to source constituents in the oil.

## Materials and Methods

### Preparation of Asphaltene and Resin Fractions

Crude oil was collected from well 301 at the Bemidji site in 1986 and 1988. The 1986 sample was found to be essentially identical in all major characteristics to a reference sample from the oil company [38]. Permission for sample collection at the Bemidji site was obtained from Beltrami County, Minnesota and the state of Minnesota [39]. Separate batches of asphaltene and resin fractions were prepared from each of the two oil samples. The asphaltene was precipitated from the crude oil with pentane using a 50:1 ratio of solvent to oil. After removal of pentane with rotary evaporation, the maltene fraction was chromatographed on silica gel (100–200 mesh) into saturate (heptane elution), aromatic (benzene elution) and resin (50:50 benzene: methanol elution) fractions. Carbon-13 NMR spectra were recorded on underivatized and methylated fractions prepared from the 1986 sample [35]. Reactions with hydroxylamine and aniline and ^15^N NMR spectra were performed on fractions prepared from the 1988 sample. Elemental analyses (Huffmann Laboratories, Golden, CO.) were performed on both sets of asphaltene and resin fractions (Table 1). The mostly minor differences in elemental analyses between the 1986 and 1988 samples are assumed to originate in the fractionation and not the contents of the two oil samples. The one significant discrepancy, the higher oxygen content of the resin from the 1988 oil sample, may be due in part to the inadvertent entrainment of silica gel within the resin fraction.

**Table 1:** Elemental analyses of resin and asphaltene fractions before and after derivatization with hydroxylamine and aniline.

Sample	C ^a^	H	N	O	S	Ash
**1986 Resin**	**80.0**	**9.6**	**1.4**	**5.1**	**2.9**	**0.3**
**1988 Resin**	**77.3**	**9.5**	**0.8**	**10.5**	**2.3**	**1.3**
**1988 Resin/NH**_**2**_ **OH**	**77.4**	**9.7**	**0.9**	**9.7**	**2.1**	**<0.5**
**1988 Resin/PhNH**_**2**_	**77.6**	**9.7**	**0.9**	**9.9**	**2.3**	**<0.5**
**1986 Asphaltene**	**86.5**	**7.2**	**1.4**	**2.6**	**3.1**	**<0.1**
**1988 Asphaltene**	**84.9**	**7.5**	**1.3**	**3.8**	**3.4**	**2.9**
**1988 Asphaltene/NH**_**2**_ **OH**	**85.3**	**7.5**	**1.5**	**2.8**	**3.4**	**0.6**
**1988 Asphaltene/PhNH**_**2**_	**85.3**	**7.4**	**1.4**	**2.9**	**3.4**	**1.1**

^a^ C, H, N, O and S reported on moisture free and ash free basis to ±0.03% accuracy as weight percent.

### Phase Transfer Catalysis Methylation of Asphaltene and Resin Fractions

The asphaltene and resin fractions were methylated using a phase transfer catalysis (PTC) procedure [40] employing ^13^CH_3_I (99 atom % ^13^C, Aldrich), tetra-n-butyl ammonium hydroxide (40%, Aldrich), and tetrahydrofuran (100 mg asphaltene or resin; 26 μL ^13^CH_3_I; 0.21 mL TBAH; 5 ml THF; 5 day reaction time). Tetra-n-butyl ammonium salts were washed from the methylated samples with aqueous sodium nitrate solutions after the reactions were quenched with 1N HCl. (Use of trade names in this report is for identification purposes only and does not constitute endorsement by the U.S. Geological Survey.)

### Diazomethylation of Asphaltene and Resin Fractions

Fifty mg of the asphaltene and 100 mg of the resin dissolved in chloroform were methylated with ^13^C-labeled diazomethane generated from 0.5 gram and 0.7 gram respectively of DIAZALD (N-methyl-^13^C-N-nitroso-p-toluenesulfonamide; 99 atom % ^13^C; Cambridge Isotope Laboratories).

### Oximation of Asphaltene and Resin Fractions

In a modification of a procedure reported by Schnitzer and Khan [41], 120 mg of ^15^NH_2_OH.HCL (99 atom % ^15^N; ISOTEC) dissolved in 20 mL dried methanol, 400 mg asphaltene dissolved in 410 mL chloroform, and 135 μL N,N-dimethylethanolamine (deanol) were added to a stoppered 1 L flask and stirred for 5 days at room temperature. The reaction flask contained an adequate headspace of air to allow for oxidation of hydroquinone and catechol moieties. The solvents were then evaporated under nitrogen. The derivatized asphaltene was redissolved in chloroform and washed with water in a separatory funnel to remove the salts and deanol. The chloroform was again evaporated under nitrogen. The resin was reacted similarly: 75 mg of ^15^NH_2_OH.HCL dissolved in 15 mL methanol; 125 mL chloroform; 250 mg resin; 84 μL deanol; 500 mL flask; 5 days. For solid state NMR analysis, the washed and dried resin was redissolved in chloroform and slurried with alumina (150 mesh, activated, weakly acidic; Aldrich) in a ratio of three parts alumina to one part resin oxime. The sample was packed into a rotor following evaporation of the solvent.

### Reaction of Asphaltene and Resin Fractions with Aniline (Schiff Base Formation)

In a 200 mL round bottom flask, 300 mg of the asphaltene or resin was dissolved in 50 mL chloroform, charged with 50 μL aniline (99 atom % ^15^N; ISOTEC), and refluxed 5 days. Samples were washed with water in a separatory funnel to remove unreacted aniline, and the chloroform was then evaporated under nitrogen.

### NMR Spectroscopy

Liquid-state NMR spectra were recorded on a VARIAN Gemini 300 MHz spectrometer at ^13^C and ^15^N resonant frequencies of 75.4 MHz and 30.4 MHz, respectively, using a 10 mm broadband probe. All samples were dissolved in deuterated chloroform. Quantitative ^13^C NMR spectra (single pulse experiment; differential T_1_ and NOE effects eliminated) of the underivatized asphaltene and resin were recorded as previously reported [35], using a 250 ppm (18,867.9 Hz) spectral window, 90° observe pulse, 0.5 sec acquisition time, 20.0 sec pulse delay and inverse gated decoupling. The pulse delay of 20 seconds is five times the longest spin lattice relaxation time, T_1_, measured for the samples and allows for complete relaxation of ^13^C nuclei between pulses, eliminating differential saturation effects. Inverse gated decoupling (gated decoupling without NOE) eliminates differential nuclear Overhauser enhancement (NOE) effects. Concentrations were 108 mg of resin and 150 mg of asphaltene in 2 mL CDCl_3_. DEPT ^13^C NMR spectra (distortionless enhancement by polarization transfer, GL version; [42]) were recorded using a 250 ppm spectral window, 0.5- sec acquisition time, and 2.0- sec delay for proton relaxation, assuming maximum and minimum values for ^1^J_CH_ of 160 and 125 Hz, respectively. DEPT ^15^N NMR spectra were recorded using an 855.3 ppm (26000 Hz) spectral width, 0.2 sec acquisition time, 1.0 sec pulse delay for proton relaxation, and ^1^J_NH_ of 90 Hz. Solid-state cross polarization/magic angle spinning (CP/MAS) ^15^N NMR spectra were recorded on a Chemagnetics CMX-200 spectrometer at 20.3 MHz using a 7.5 mm ceramic probe (zirconium pencil rotors) and constant amplitude cross polarization. Acquisition parameters included a 1315.3 ppm spectral window (26.0 KHz), 17.051 msec acquisition time, 0.2 sec pulse delay, and spinning rate of 5 or 6 KHz. (A comparison of spectra recorded at longer pulse delays of from 0.5 to 1.0 sec with those recorded at 0.2 sec showed no discernible differences.) Contact times were chosen to provide adequate signal intensities for all features of interest in each sample: 2.0 msec in the underivatized asphaltene to achieve a reasonable signal to noise ratio of naturally abundant nitrogens while providing some allowance for more slowly polarizing nuclei; 1.0 msec for the hydroxylamine derivatives to enhance signal intensities of secondary reaction products; 5.0 msec for the aniline derivative of the asphaltene to enhance intensities of imine nitrogens (vide infra). Nitrogen-15 NMR chemical shifts are reported in ppm downfield from ammonia, taken as 0.0 ppm. (Note: The ^15^N NMR spectrum of the resin oxime was recorded at a spinning rate of 5 kHz; at a spinning rate of 6 kHz some resin was observed to leak from the rotor.) More detailed background information on the solid state ^15^N CP/MAS experiment is available [43–45]. Carbon-13 and ^15^N NMR chemical shifts reported here are accurate to ± 0.5 ppm.

## Results

### ^13^C NMR Spectra of Resin and Asphaltene Fractions Before and After Methylation

**Fig 1:**
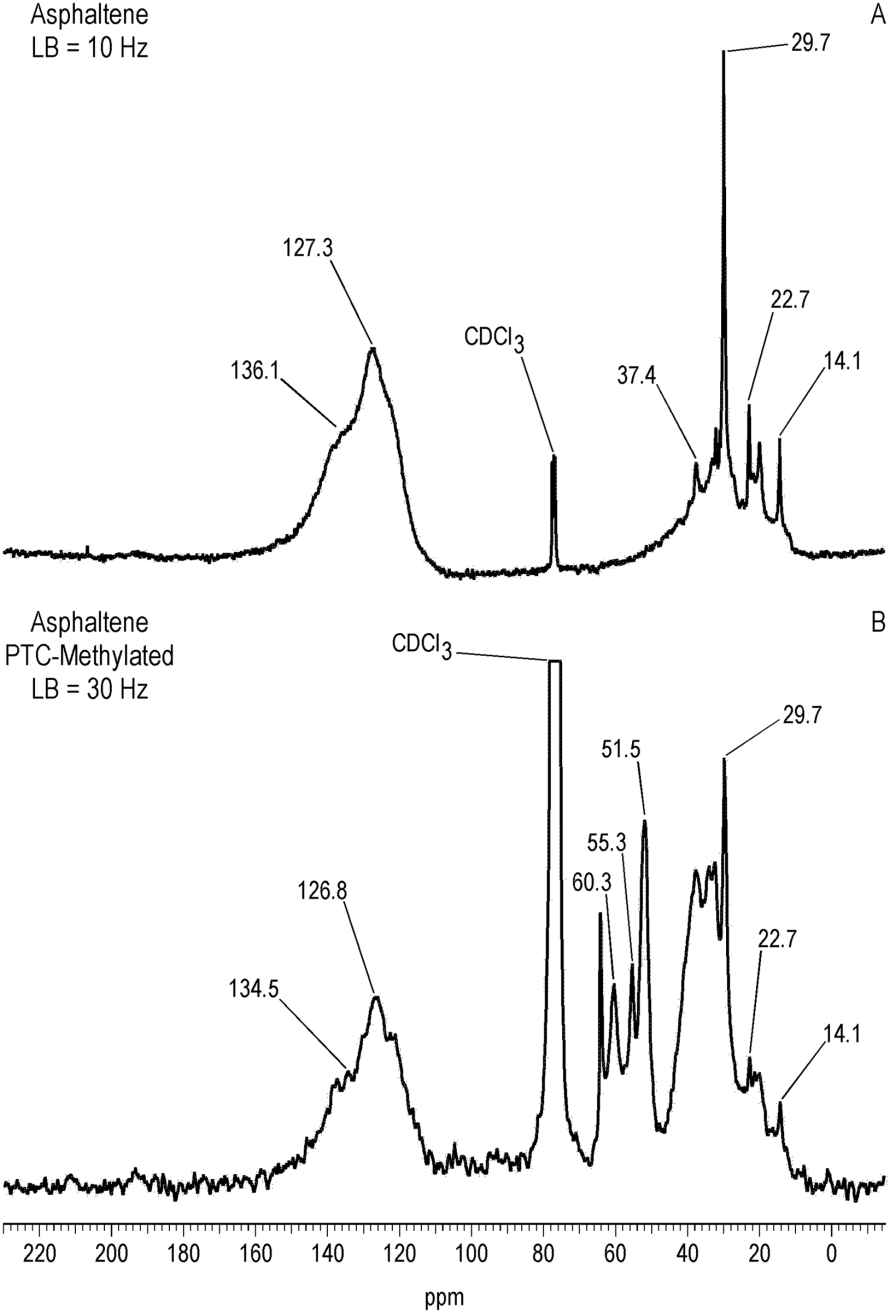
Quantitative liquid-state C-13 NMR spectra of asphaltene before and after PTC-methylation with ^13^CH_3_I. LB = line broadening. For the underivatized asphaltene, carbon aromaticity(f_a_) = 0.63.

**Fig 2:**
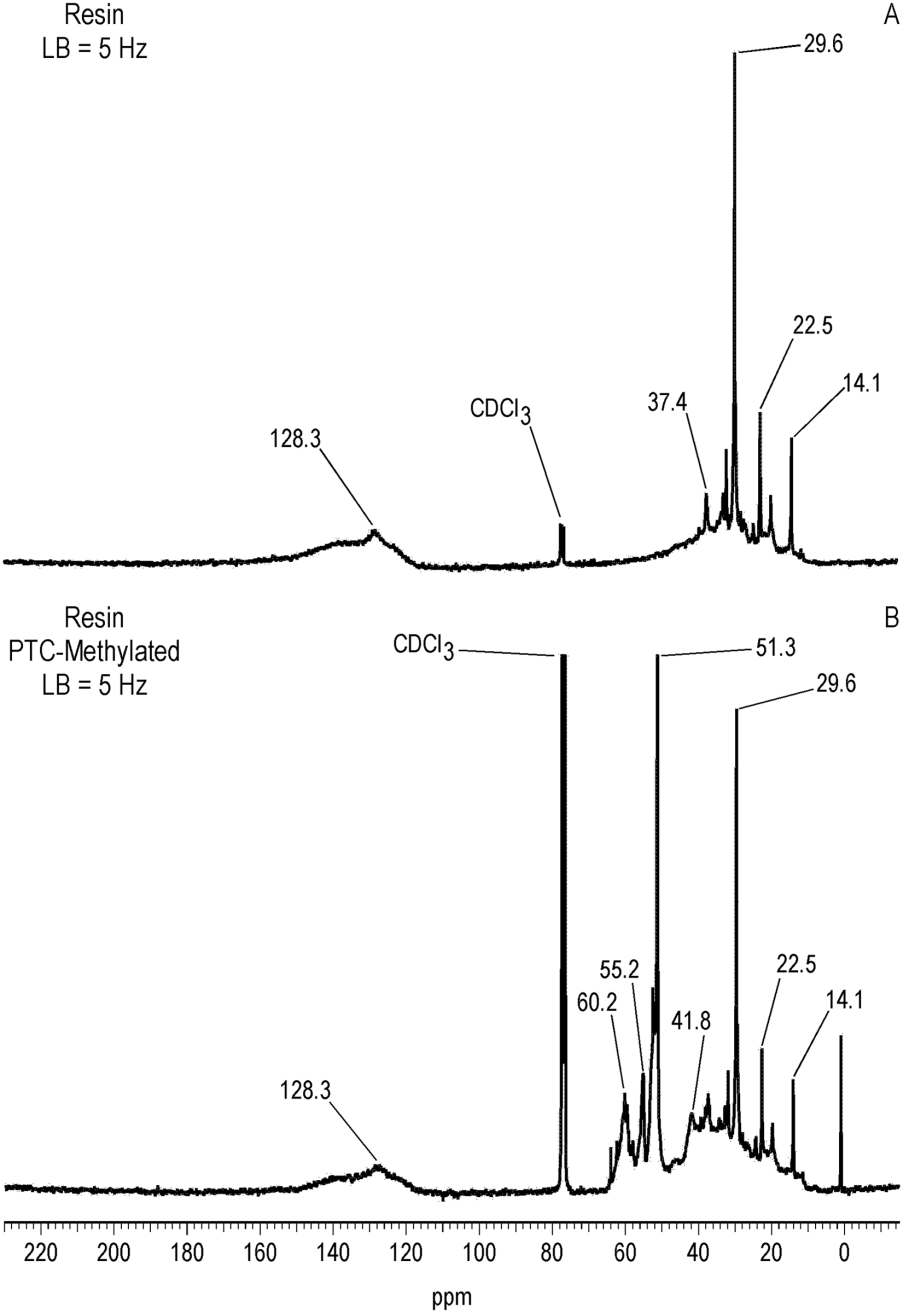
Quantitative liquid-state C-13 NMR spectra of resin before and after PTC-methylation with ^13^CH_3_I. LB = line broadening. For the underivatized resin, carbon aromaticity(f_a_) = 0.33.

**Fig 3:**
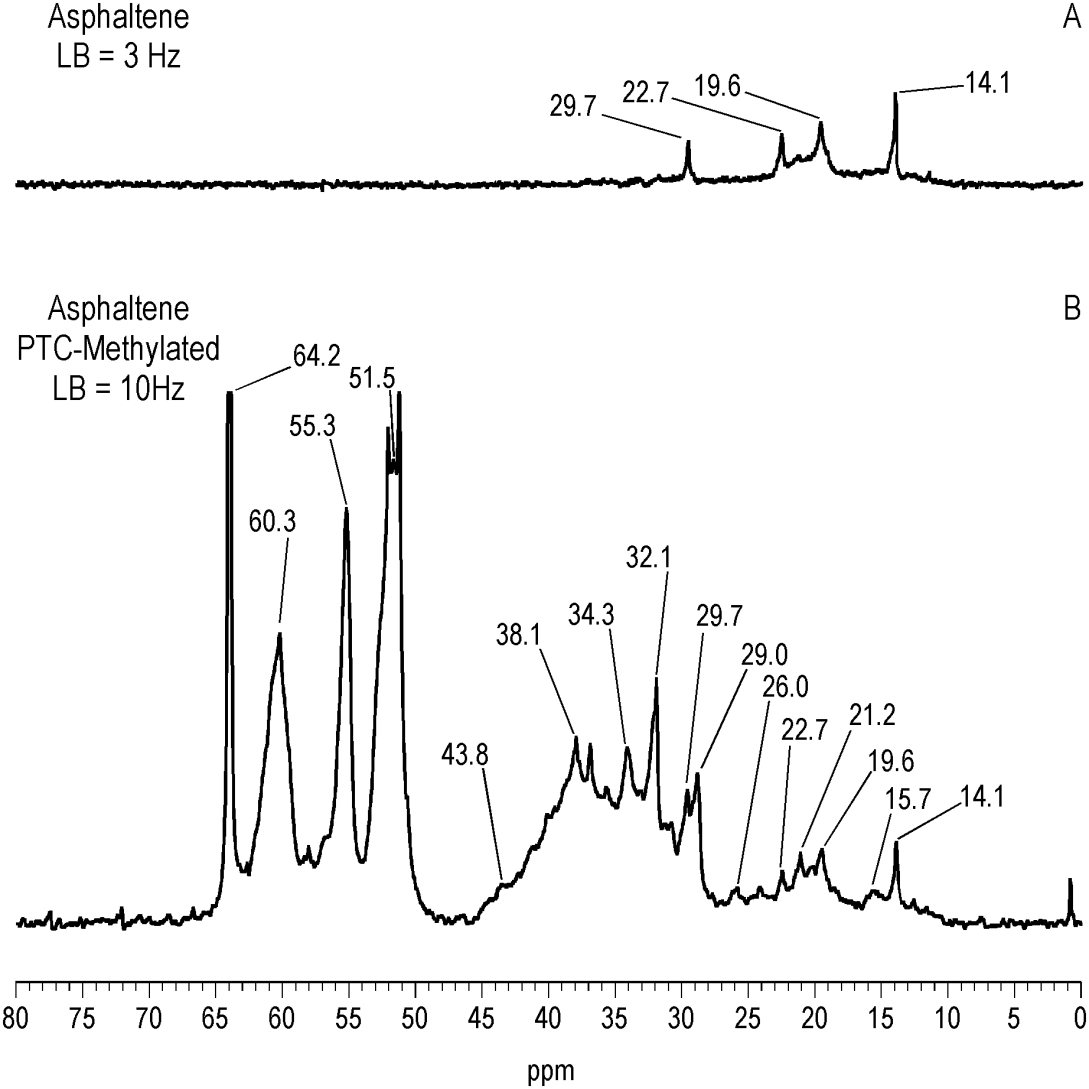
Liquid-state DEPTGL C-13 NMR methyl subspectra of asphaltene before and after PTC-methylation with ^13^CH_3_I. LB = line broadening.

**Fig 4:**
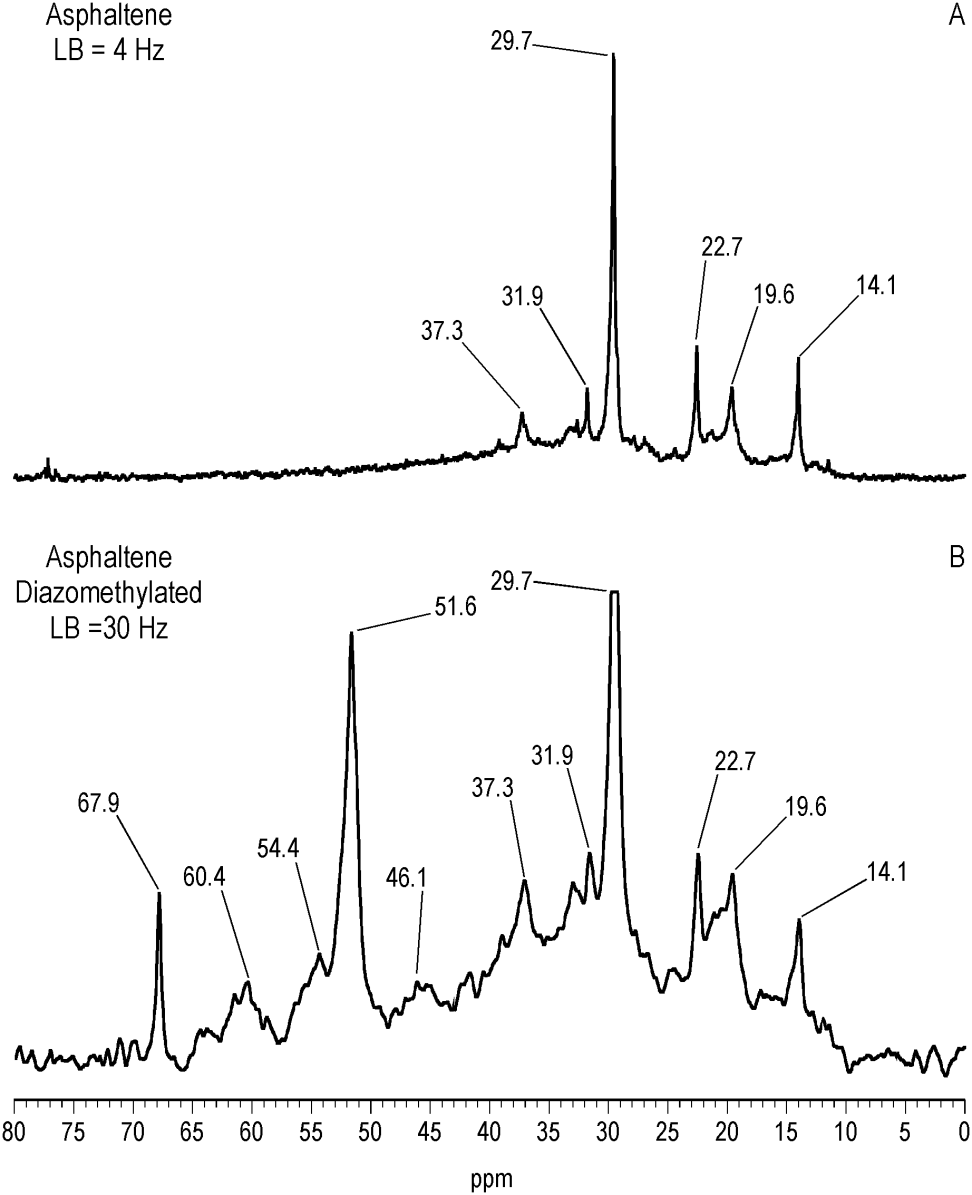
Liquid-state DEPTGL C-13 NMR spectra showing all protonated carbons of asphaltene before and after ^13^C-diazomethylation. LB = line broadening.

**Fig 5:**
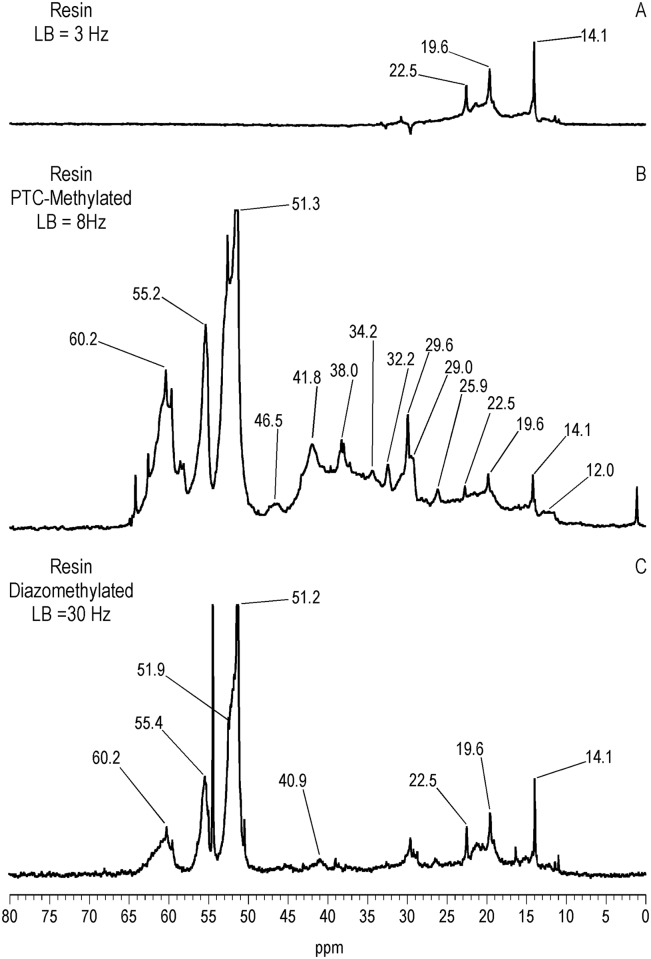
Liquid-state DEPTGL C-13 NMR methyl subspectra of resin before and after PTC-methylation with ^13^CH_3_I and ^13^C-diazomethylation. LB = line broadening.

**Fig 6:**
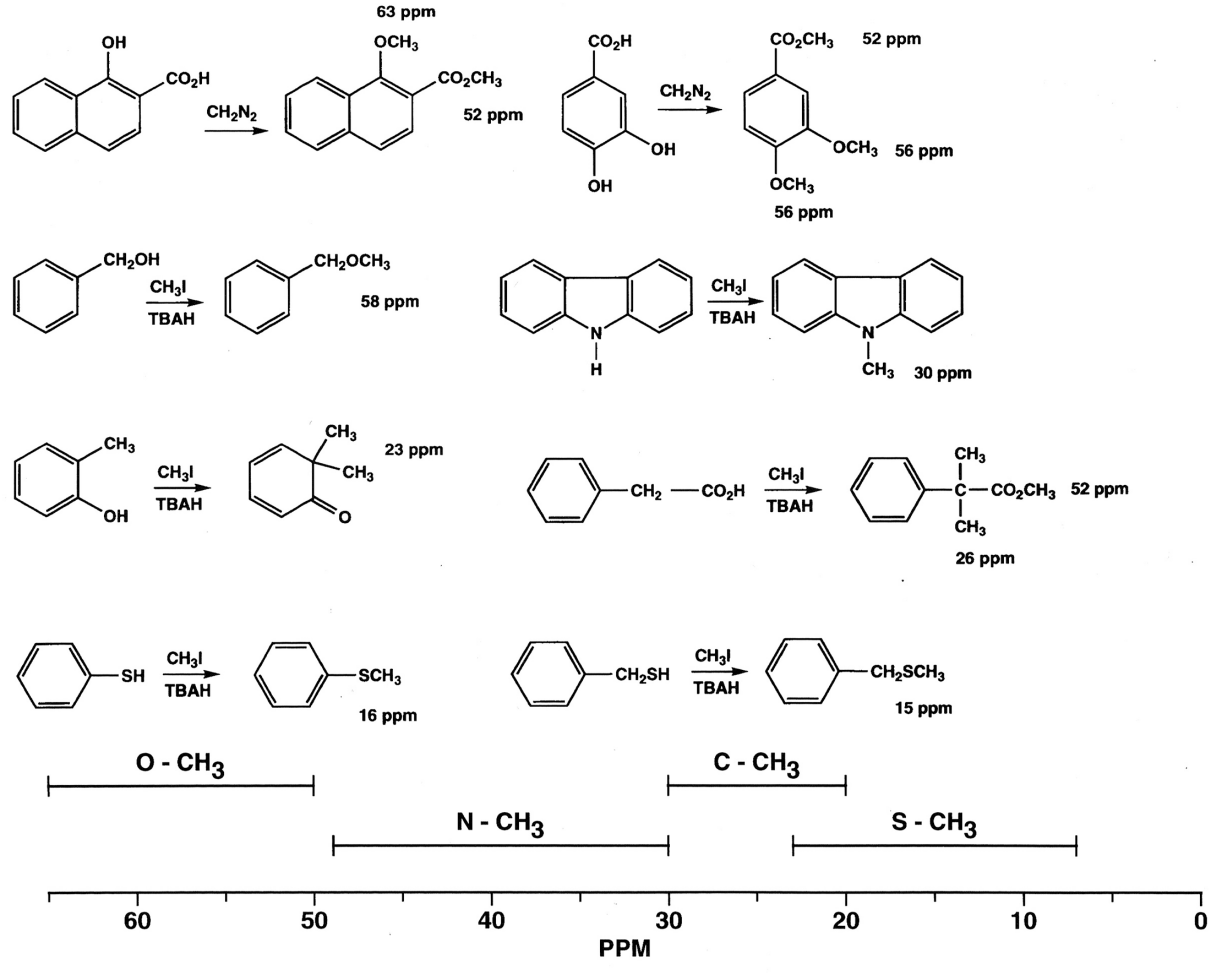
Carbon-13 NMR chemical shift ranges and examples of O-CH_3_, N-CH_3_, C-CH_3_ and S-CH_3_ methyl carbons resulting from methylation of acidic oxygen, carbon, nitrogen and sulfur groups.

Characteristically of underivatized asphaltenes and resins reported in the literature, the quantitative liquid-state ^13^C NMR spectra show aliphatic carbons from 10 to 60 ppm and aromatic carbons from 115 to 155 ppm (Figs 1A and 2A), with the asphaltene having a greater carbon aromaticity than the resin (f_a_ = 0.63 and f_a_ = 0.33, respectively) [35]. More detailed assignments can be found in the extensive ^13^C NMR chemical shift compilations of Altgelt and Boduszynski [1]. Carbons corresponding to carboxylic acids, ketones, quinones and other carbonyl groups (165 to 230 ppm) are not visible in the spectra and thus below the detection limit of the ^13^C NMR experiment at the concentrations, field strength and number of transients employed. The PTC procedure methylates acidic oxygen, nitrogen, carbon and thiol sulfur (aliphatic and aromatic mercaptans), but not thiophene sulfur [40,46]. These functional groups are visualized via their labeled methyl derivatives in the quantitative ^13^C NMR spectra of Figs 1B and 2B, and most clearly in the DEPTGL subspectra of Figs 3–5. Carbon-13 NMR chemical shift ranges for O-CH_3_, N-CH_3_, C-CH_3_ and S-CH_3_ groups are illustrated in Fig 6. Focusing on the methyl carbon subspectra of the PTC-methylated samples (Figs 3B and 5B), the major peak at 51 ppm corresponds to the methyl esters of carboxylic acids, whereas the peaks at 55 and 60 ppm correspond to the methyl ethers of alcohol and phenolic hydroxyls. The chemical shift range for N-CH_3_ groups is approximately from 50 to 25 ppm, and for C-CH_3_ groups from 30 to 20 ppm. Both the asphaltene and resin exhibit significant N-methylation, with peaks at 38.1 and 34.3 ppm in the asphaltene, and 46.5, 41.8 and 34.2 ppm in the resin. These peaks may include N-CH_3_ derivatives of amides and amines, with N-methyl derivatives of carbazole nitrogens occurring at about 30 ppm. Spectral integrations indicate that concentrations of acidic nitrogens (50 to 30 ppm) are approximately 78% of the acidic oxygens (65 to 50 ppm) in the asphaltene and 59% of the acidic oxygens in the resin. Methylation of carbon and sulfur occurs to a lesser degree than oxygen and nitrogen, as the peaks upfield from about 30 ppm correspond mainly to the background carbons of the underivatized samples, at 29.7 (incompletely cancelled methylene peak), 22.7, 19.6 and 14.1 ppm in the asphaltene, and 29.6 (incompletely cancelled methylene peak), 22.5, 19.6 and 14.1 ppm in the resin. Peaks at 29.0 and 26.0 ppm in the asphaltene and 29.0 and 25.9 ppm in the resin could be N-CH_3_ or C-CH_3_ groups. The peak at 21.2 ppm in the asphaltene can be attributed to a C-CH_3_ group. Minor peaks attributable to sulfur methylation (S-CH_3_) occur at 15.7 ppm in the asphaltene and 12.0 ppm in the resin. Integration of the methyl ester and ether peaks against the aromatic carbon peaks in the quantitative ^13^C NMR spectra (Figs 1B and 2B) in conjunction with elemental analyses of the underivatized samples (1986 values) yields estimates of 0.39 mmol/gram and 0.28 mmol/gram for the total hydroxyl group concentrations of the asphaltene and resin, respectively. These values are within the range of total hydroxyl group concentrations reported for a series of asphalts methylated using the PTC procedure [30]. During the course of the PTC procedure it is possible that ester groups become hydrolyzed and the resulting carboxylic acid and alcohol groups undergo methylation. Hydrolysis is not a concern during methylation with diazomethane, which, for acidic oxygens, is selective for carboxylic acid and phenolic hydroxyl groups. In the ^13^C NMR spectra of the diazomethylated samples (Figs 4B and 5C), methyl esters of carboxylic acids occur at 51.6 ppm in the asphaltene and 51.9 ppm in the resin. Methyl ether peaks of phenolic hydroxyls, including catechol and hydroquinone moieties that can potentially oxidize to quinones, occur at 54.4 and 60.4 ppm in the asphaltene and at 55.5 and 60.2 ppm in the resin. The downfield peaks at 60.4 and 60.2 ppm furthermore correspond to the methyl ethers of phenolic hydroxyls adjacent to two substituents, where the ring juncture of a condensed aromatic structure counts as a substituent. Quinones that oxidize from catechol or hydroquinone moieties are potential substrate sites for reaction with hydroxylamine and aniline. Some nitrogen groups are also susceptible to derivatization with diazomethane, indicated by the minor N-methyl peaks at 46.1 ppm in the asphaltene and 40.9 ppm in the resin.

### Natural Abundance ^15^N NMR Spectrum of Asphaltene Fraction

**Fig 7:**
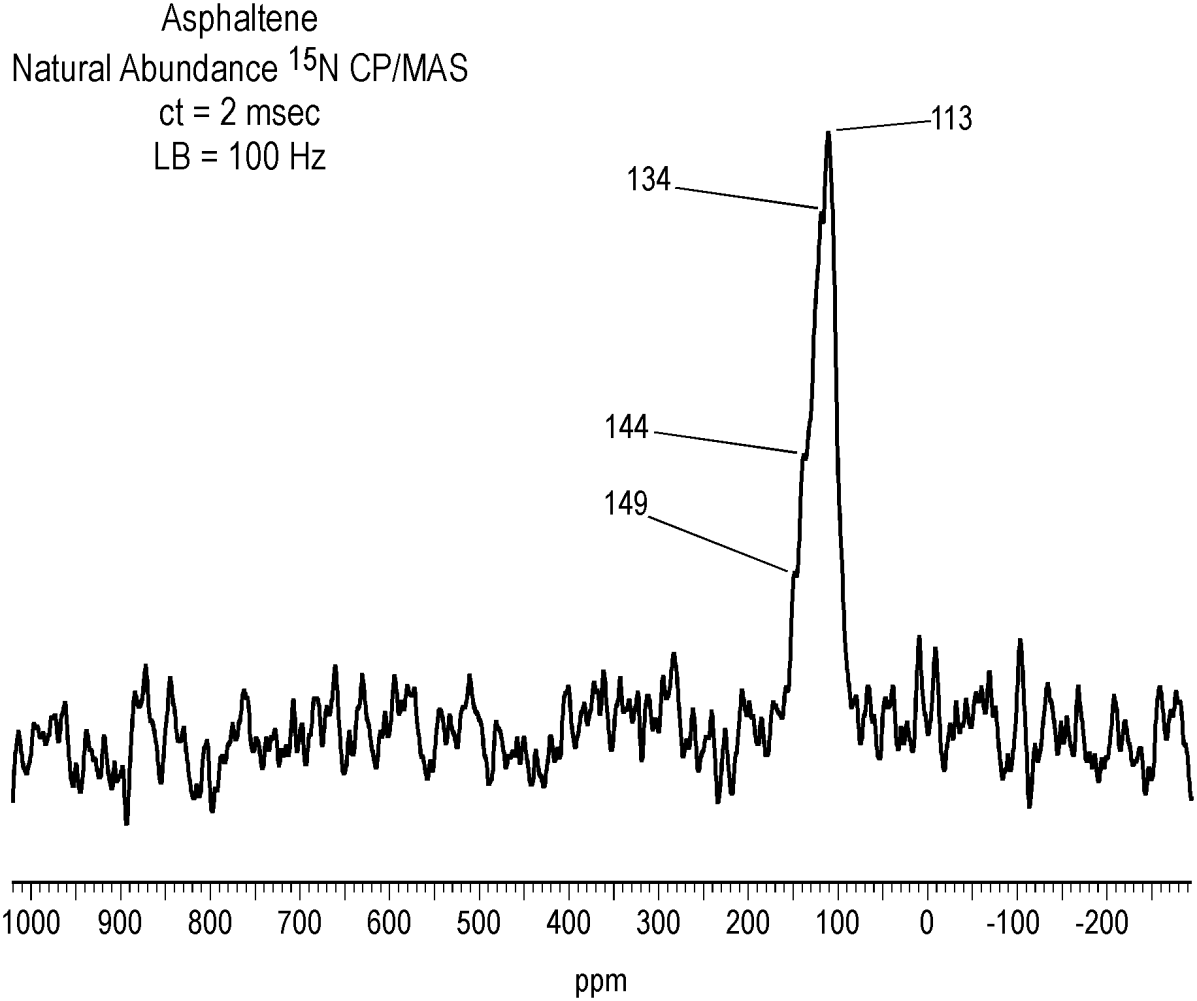
Solid-state CP/MAS N-15 NMR spectrum of naturally abundant asphaltene nitrogen. LB = line broadening. ct = contact time. Spinning speed = 5 kHz.

Altgelt and Boduszynski [1] found it useful to classify nitrogen in heavy petroleum fractions as basic (pyridine, primary amine, N-alkylindole, alkyl-arylamine), neutral (alkylindole, alkylacridine, certain amides, alkylhydroxypyridines) and acidic (indoles, carbazoles, nonmetallic porphyrins and amides). A number of studies have concluded that pyrrolic nitrogen is the major form in asphaltenes [2,4]. This appears to be the case in the Bemidji asphaltene, where the peak maximum at 113 ppm in the solid-state CP/MAS ^15^N NMR spectrum of the naturally abundant nitrogen corresponds to carbazole nitrogen (Fig 7). The broad peak from about 95 to 155 ppm also encompasses other pyrrole-like nitrogen heterocyclic structures such as indoles and the pyrrole-type nitrogens of porphyrins, in addition to quinolone and amide nitrogens. The spectrum is similar to one reported for gilsonite, a naturally occurring bituminous asphalt [47]. Pyridine (~ 270–330 ppm) and primary amine (~ 0 to 75 ppm) nitrogens are not observed in the spectrum of Fig 7. In the case of pyridine nitrogens, including the pyridine-like nitrogens of porphyrins (~220–300 ppm), factors such as unfavorable spin dynamics (e.g. slow polarization of nitrogens not directly bonded to protons), the large chemical shift anisotropy of pyridine-like nitrogens, and chemical exchange phenomena may impede detection [47–51]. Or, more fundamentally, in analogy to the fact that carbonyl groups are below the detection limit in natural abundance ^13^C NMR spectra of asphaltenes and resins at commonly used acquisition parameters, some types of asphaltene nitrogens may also lie below the detection limit of the solid state experiment. For the spectrum presented here, a contact time of 2 msec was chosen to achieve a reasonable signal-to-noise ratio per unit of spectrometer time while allowing, to some extent, for detection of more slowly polarizing nuclei, but a longer contact time may be necessary. X-ray absorption near-edge structure (XANES) spectroscopy has indicated the presence of both basic pyridinic and acidic pyrrolic nitrogen in asphaltenes [52]. The detection of pyridine nitrogen by XANES but not ^15^N NMR is a discrepancy that has also been noted for soil and aquatic humic and fulvic acids. Pyridine nitrogens have been inferred from both XANES and XPS (X-ray photoelectron spectroscopy) studies of humic substances but have not been observed in ^15^N NMR spectra [53]. In short, although undetected in the ^15^N NMR spectrum, the occurrence of pyridine nitrogens in the Bemidji asphaltene cannot be ruled out.

In the ^15^N NMR spectra of the derivatized asphaltenes that follow, the naturally abundant nitrogens do not contribute to the intensities of the peaks corresponding to the labeled nitrogens incorporated into the samples, in their region of overlap (95 to 155 ppm), because the signal from the label overwhelms the naturally abundant nitrogens. This can be ascertained from the relative number of transients required for both sets of signals to come up. Although a natural abundance ^15^N spectrum was not obtained, the same constraint is assumed to pertain to the resin, taking into account that it has a lower N content than the asphaltene.

### ^15^N NMR Spectra and Elemental Analyses of Oximated Asphaltene and Resin Fractions

**Fig 8:**
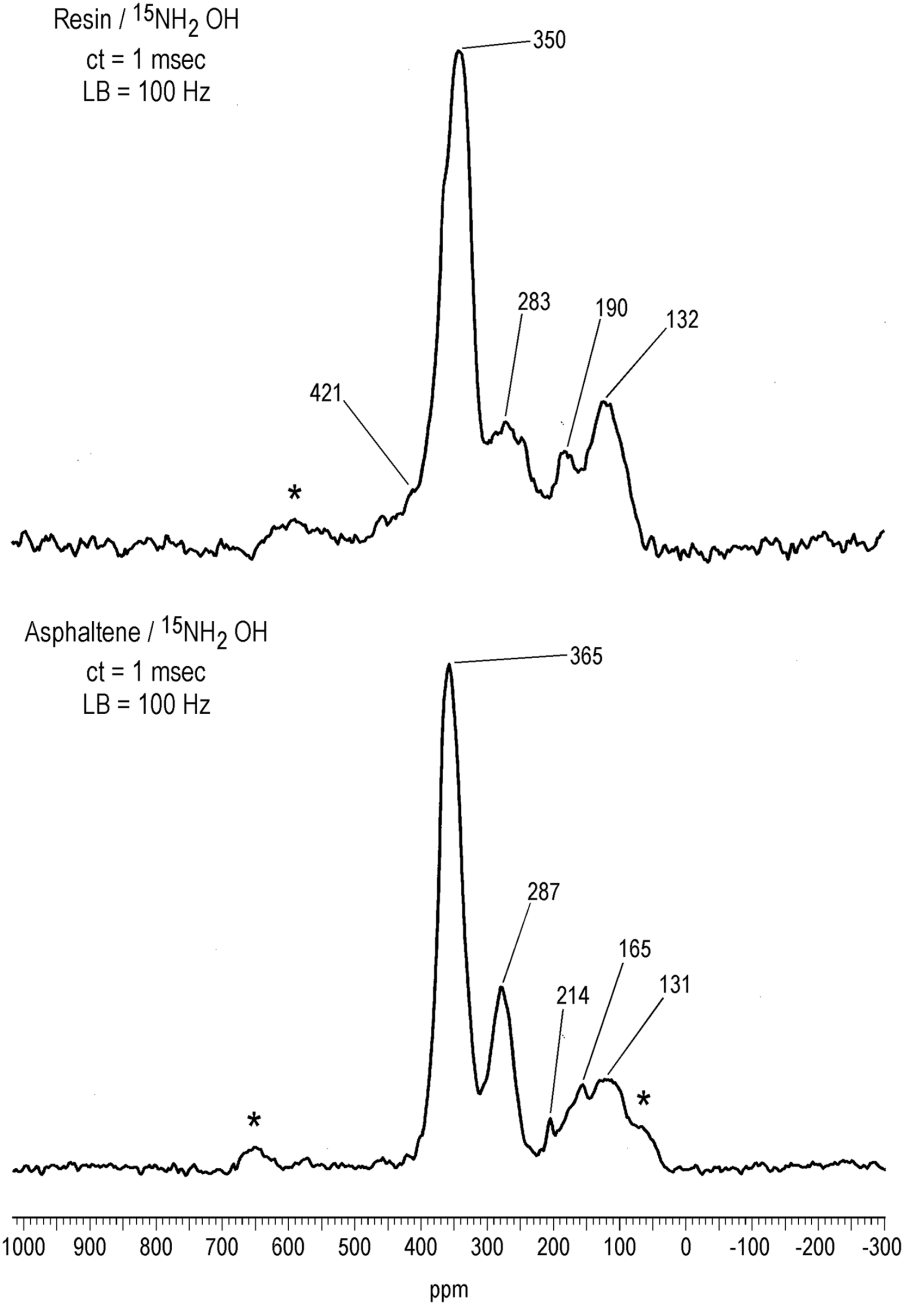
Solid-state CP/MAS N-15 NMR spectra of asphaltene and resin derivatized with ^15^N-labeled hydroxylamine. LB = line broadening. ct = contact time. Spinning speed = 5 kHz for resin and 6 kHz for asphaltene. Asterisks denote spinning sidebands.

**Fig 9:**
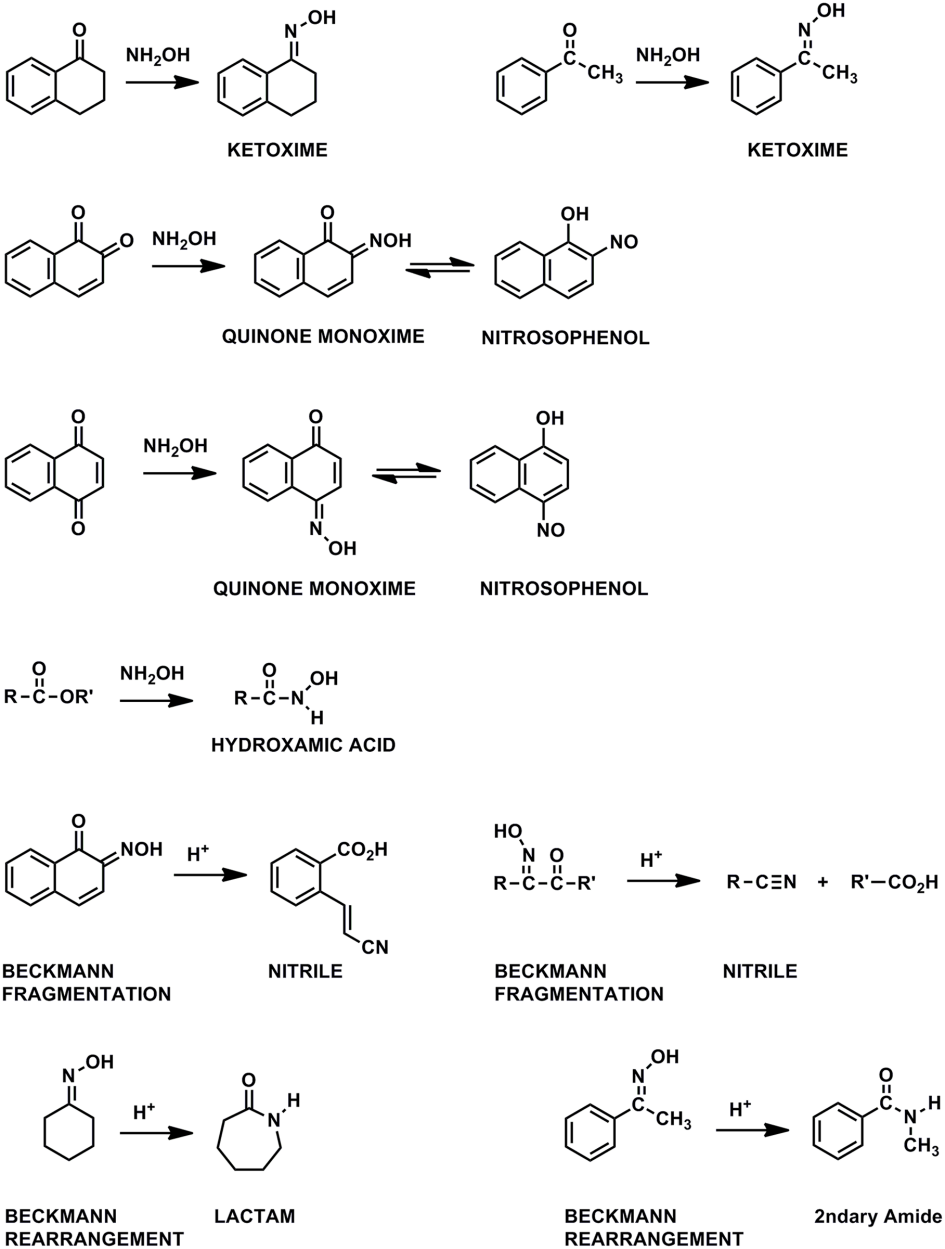
Reactions of carbonyl groups with hydroxylamine.

**Fig 10:**
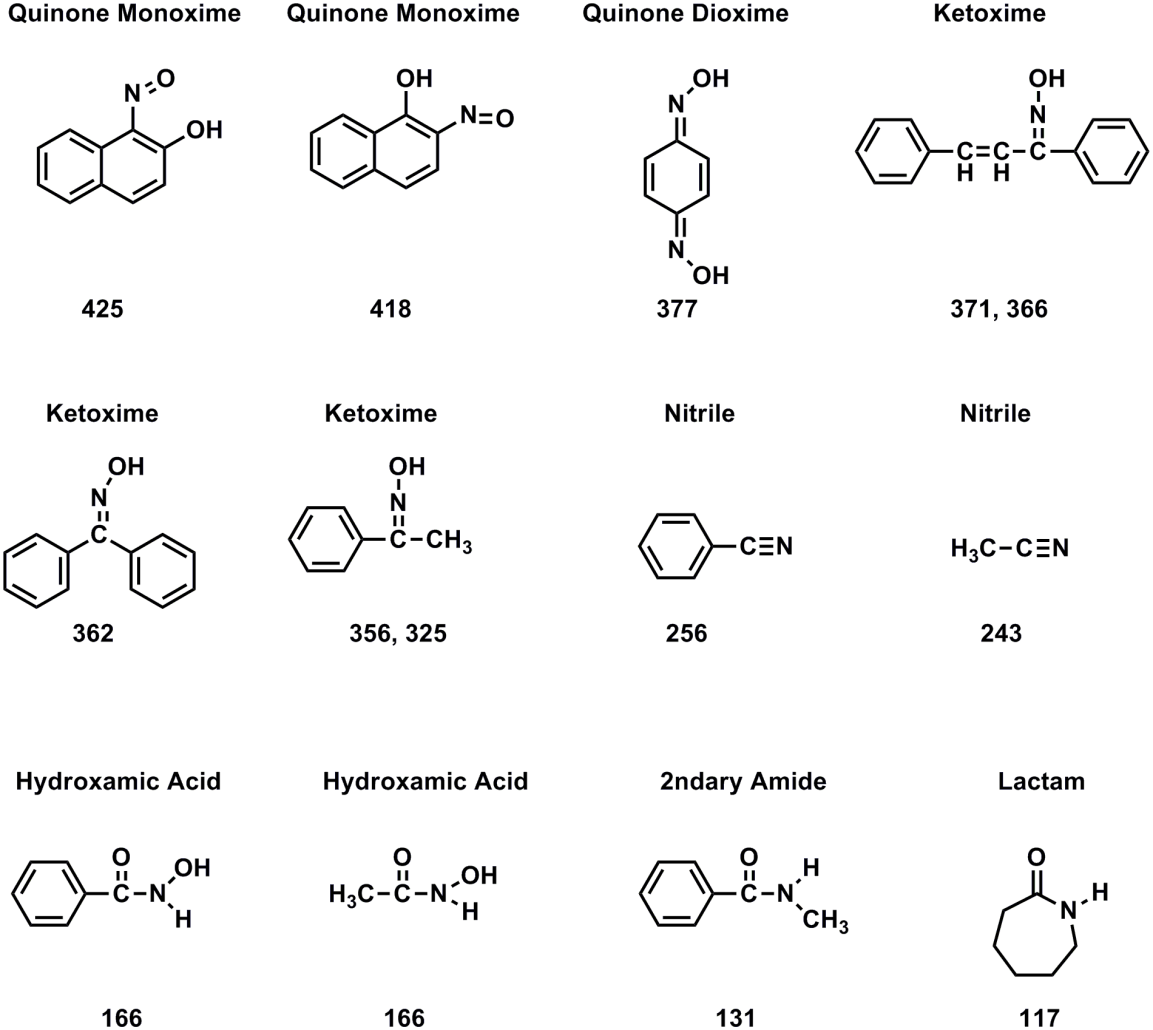
Nitrogen-15 NMR chemical shifts in ppm for oximes and Beckmann reaction products. From reference {Thorn, 1992 #523}. Separate resonances are observed for the Z and E isomers of ketoximes. In general, the E isomers of ketoximes are deshielded with respect to the Z isomers.

The main peaks in the solid-state CP/MAS ^15^N NMR spectra of the asphaltene and resin samples reacted with hydroxylamine, at 365 ppm and 350 ppm respectively, correspond to the oxime derivatives of ketones, thereby confirming the presence of ketones in these crude oil fractions (Figs 8–10). In liquid-state ^15^N NMR spectra of humic substances reacted with hydroxylamine, ketoxime peaks (330 to 385 ppm) are resolved from peaks corresponding to the mono-oxime derivatives of quinones (385 to 430 ppm) [34]. This is not the case in the solid state spectra of the asphaltene and resin oximes. The downfield (deshielded) region of the asphaltene and resin oxime peaks may contain contributions from quinone mono-oxime nitrogens, but more definitive confirmation of quinones comes from derivitization with aniline, discussed in the next section.

The remaining peaks in the spectra of Fig 8 can mainly be assigned as products that result from Beckmann reactions of the initially formed oximes (Fig 9). Nitriles and primary amides result from Beckmann *fragmentations* of oximes; lactams and secondary amides result from Beckmann *rearrangements* of oximes. Lactams arise specifically from Beckmann rearrangements of cyclic ketones. Nitriles occur at 287 ppm in the asphaltene and 283 ppm in the resin. The largest class of oximes that undergo fragmentation are oximes that have quaternary carbon centers adjacent to the oxime carbon, because of the stability of the carbonium ion that is cleaved. Examples include oximes of α -diketones, α-keto acids, α -hydroxy ketones, mono-oximes of quinones, etc. The peaks at 131 ppm in the asphaltene and 132 ppm in the resin correspond to lactams and secondary amides. Oximes of aromatic ketones are among the classes documented to undergo the rearrangement to secondary amides. The lactam/secondary amide peaks occur within a broad set of resonances encompassing the chemical shift range from approximately 70 to 230 ppm. Assignments for the downfield region from about 180 to 230 are uncertain, but may be comprised of imidate, amidine, imidazole or isocyanide nitrogens [31]. In the asphaltene spectrum, the resolved peak at 165 ppm corresponds to hydroxamic acids that result from reaction of hydroxylamine with ester groups. A resolved hydroxamic acid peak is not present in the solid state spectrum of the resin, but was inferred from a liquid-state DEPT ^15^N NMR spectrum recorded on the sample (not shown). Assignments for the spectra of the oximated samples are summarized in Table 2.

**Table 2:** Assignments for N-15 NMR spectra of resin and asphaltene fractions derivatized with hydroxylamine.

Chemical Shift of Peak Maximum (ppm)		Chemical Shift Range (ppm)	Assignment	
Asphaltene	Resin			
**365**	**350**	**430–330**	**Oxime**	
		**430–390**	**Quinone Monoxime**	
		**390–330**	**Ketoxime**	
**287**	**283**	**320–235**	**Nitrile**	
		**230–70**	**Secondary Reaction Products**	
**214**	**190**	**230–180**	**Imidate**	**Amidine**
			**Imidazole**	**Isocyanide**
**165**		**170–160**	**Hydroxamic Acid**	
**132**	**131**	**150–110**	**2° Amide**	
		**120–90**	**Lactam**	**1° Amide**

Attenuated total reflectance FTIR spectra were recorded on the asphaltene and resin fractions and their oxime derivatives (spectra not shown). There were no discernible differences between the derivatized and underivatized spectra, an indication of the limitations in sensitivity of infrared spectroscopy for detection of the low concentrations of carbonyl groups that react with hydroxylamine, and an illustration of the utility of the ^15^N NMR approach.

The nitrogen contents of the asphaltene and resin fractions increase from 1.3 to 1.5% and from 0.8 to 0.9% after derivatization with hydroxylamine (Table 1). Assuming an increase of one mole nitrogen per mole of carbonyl derivatized, the carbonyl contents (ketone + quinone + ester) are estimated at 0.14 mmole/gram and 0.07 mmole/gram, respectively, for the asphaltene and resin. Due to the inability of ^13^C NMR and FTIR to detect changes in the carbonyl functionality upon reaction with hydroxylamine, complete derivatization of the carbonyl functionality with hydroxylamine cannot be verified or assumed. In the case of humic and fulvic acids, ^13^C NMR analyses indicated incomplete derivatization of ketone groups with hydroxylamine [31]. The values of 0.14 and 0.07 mmole/gram are comparable in magnitude to the range of 0.20 to 1.66 mmol/gram of carbonyl groups reported in the asphaltenes and preasphaltenes isolated from lignite coal liquefaction products, also calculated through increases in elemental nitrogen contents upon oximation [54].

### ^15^N NMR Spectra of Asphaltene and Resin Fractions Reacted with Aniline

**Fig 11:**
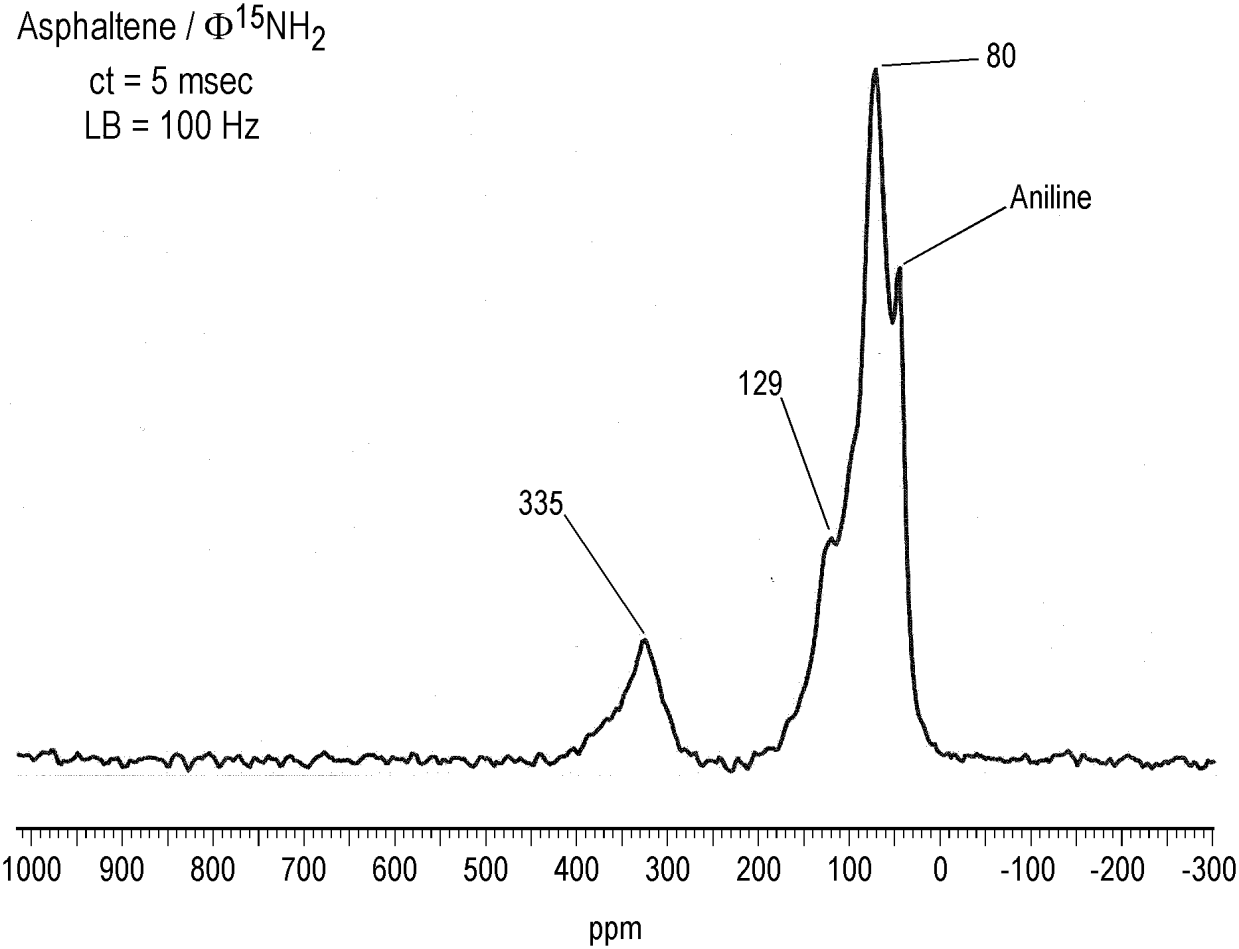
Solid-state CP/MAS N-15 NMR spectrum of asphaltene derivatized with ^15^N-labeled aniline. LB = line broadening. ct = contact time. Spinning speed = 6 kHz.

**Fig 12:**
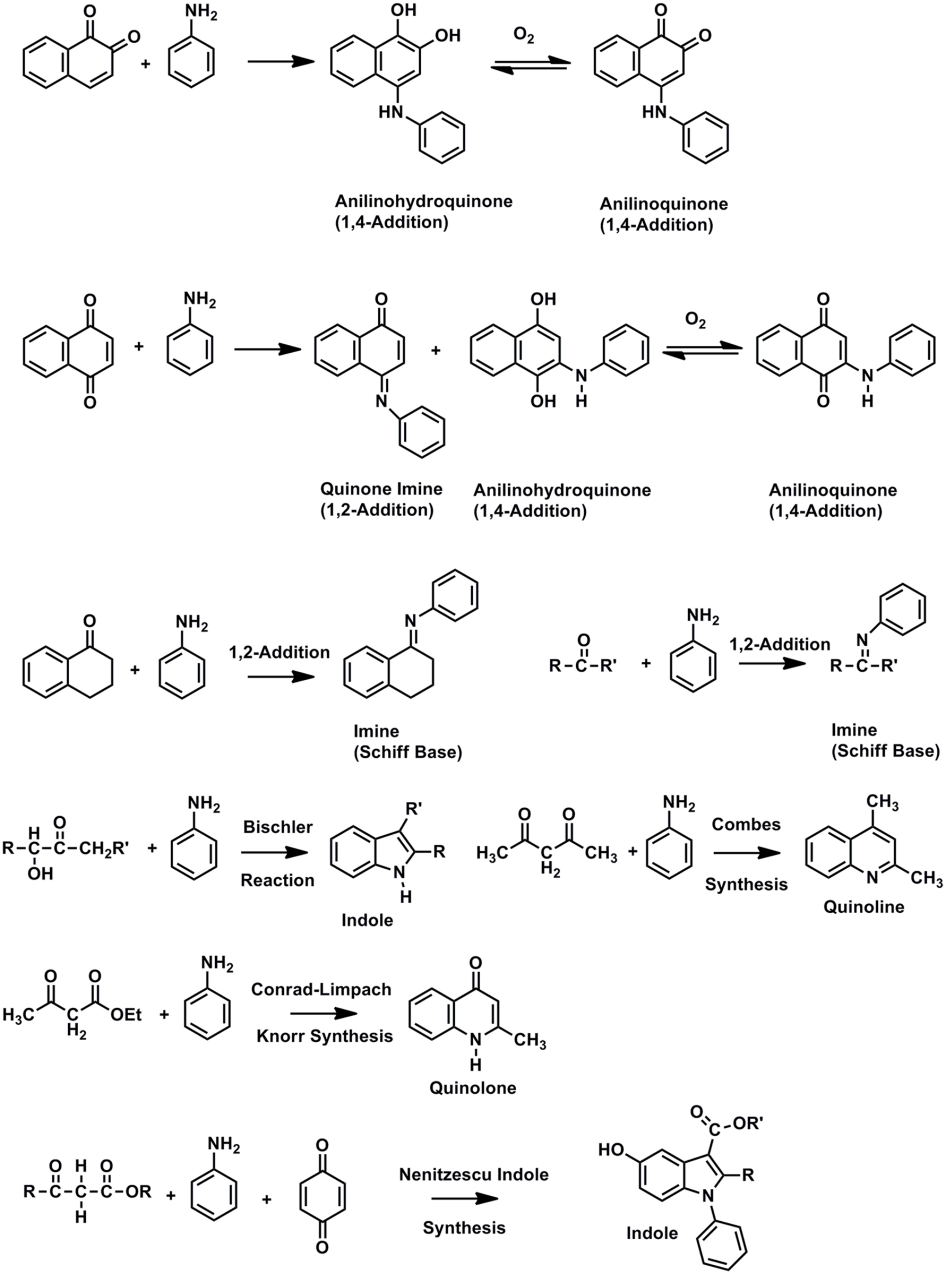
Reactions of carbonyl groups with aniline.

**Fig 13:**
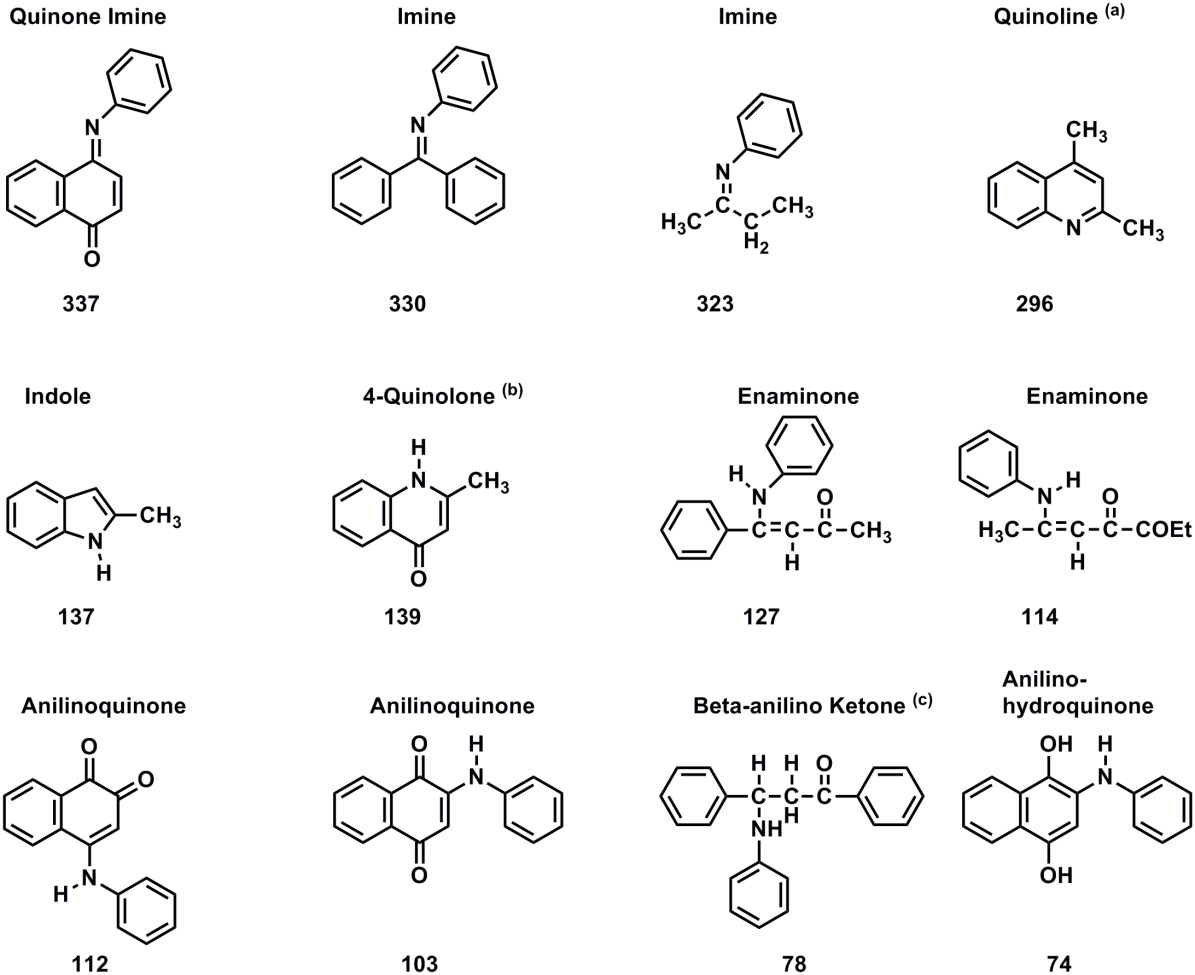
Nitrogen-15 NMR chemical shifts in ppm for Schiff Bases and condensation products of aniline with carbonyl compounds. From reference {Thorn, 1996 #277}. (a) Determined in CDCl_3_, referenced to neat aniline as 55.7 ppm. (b) Determined in CD_3_OD, referenced to neat formamide as 112.4 ppm. (b) Determined in DMSO-d_6_, referenced to neat formamide as 112.4 ppm.

The solid-state ^15^N CP/MAS spectrum of the asphaltene reacted with aniline exhibits peaks at 80 ppm, 129 ppm and 325 ppm (Fig 11). (The residual aniline occurs at 54 ppm; it was removed from the sample prior to the elemental analysis determination.) Assignments are summarized in Table 3, and reactions of aniline with carbonyl groups and ^15^N NMR chemical shifts of reaction products are illustrated in Figs 12 and 13, respectively. The peak at 80 ppm is assigned as anilinohydroquinone nitrogen, the adduct from 1,4-addition (Michael addition) of aniline to quinones. A less likely but possible assignment would be β-anilino carbonyl nitrogens from 1,4-addition (aza-Michael addition) to α, β unsaturated ketones. The peak at 335 ppm corresponds primarily to imine nitrogens, from 1,2-addition to ketones and quinones (Schiff base formation), but may also include quinoline nitrogens, that could result from the condensation of aniline with β-diketones, as in the Combes synthesis [55]. The peak at 129 ppm most likely corresponds to heterocyclic nitrogens such as indoles and quinolones, that result from intramolecular condensation reactions of aniline with ketone groups, and enaminone nitrogens, again from condensation of aniline with β-diketones. Examples of the former include the condensation of aniline with α-hydroxy ketone groups to form indoles, as in the Bischler reaction, and condensation of aniline with β-keto ester groups to form quinolones, as in the Conrad-Limpach Knorr (CLK) synthesis [55]. Peaks in this region in spectra of humic substances reacted with aniline were previously assigned primarily as anilide nitrogen [32]. Upon reconsideration of the relative resistance of ester groups to nucleophilic substitution by aromatic amines (compared to hydroxylamine and ammonia, for example), the assignment as heterocylic nitrogen appears more reasonable. The downfield portion of the peak centered at 129 ppm that extends to approximately 190 ppm may be comprised of heterocyclic nitrogens such as N-substituted indoles. These could arise from the intermolecular condensation of aniline with a β–keto ester and quinone as in the Nenitzescu indole synthesis [55].

**Table 3:** Assignments for N-15 NMR spectra of asphaltene derivatized with aniline.

Chemical Shift of Peak Maximum (ppm)	Chemical Shift Range (ppm)	Assignment			
**335**	**400–290**	**Imine**	**Quinoline**		
**129**	**190–120**	**Heterocyclic Nitrogen**	**Quinolone**	**Indole**	**Enaminone**
	**120–100**	**Anilinoquinone**	**Enaminone**		
	**100–60**	**Anilinohydroquinone**	**β-anilino Ketone**		
**80**		**Anilinohydroquinone**			

**Fig 14:**
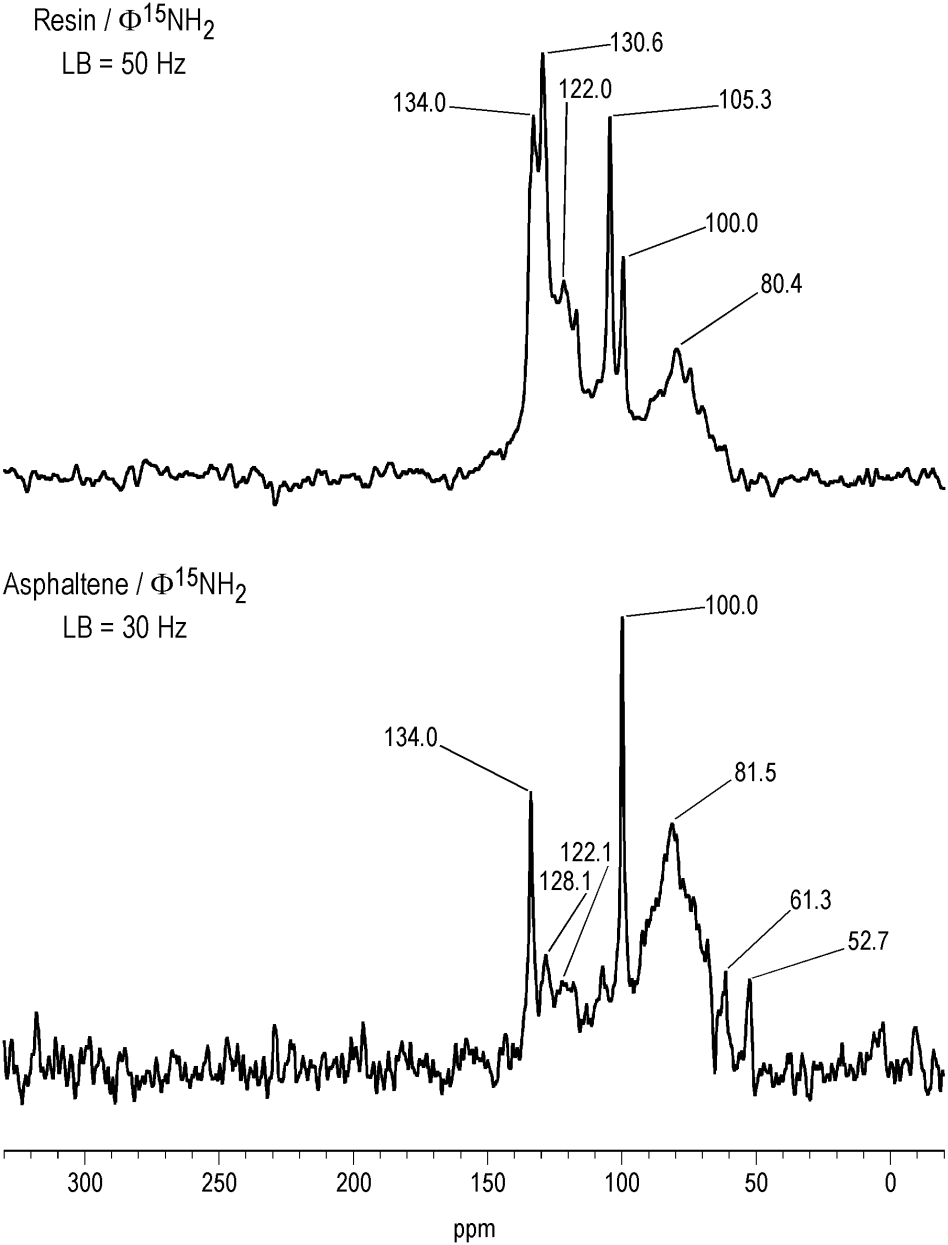
Liquid-state DEPT N-15 NMR spectra of asphaltene and resin derivatized with ^15^N-labeled aniline. LB = line broadening.

The DEPT ^15^N NMR spectra of the asphaltene and resin fractions reacted with aniline show only nitrogens directly bonded to protons (Fig 14). Prominent anilinohydroquinone peaks are present at 81.5 ppm and 80.4 ppm in the asphaltene and resin, respectively. Several discrete peaks are resolved in the liquid spectra, superimposed upon the three main regions, from approximately 60 to 95 ppm, 95 to 115 ppm, and 115 to 145 ppm, corresponding to the anilinohydroquinone, anilinoquinone and heterocyclic nitrogens, respectively. The sharp peaks, at 100.0, 105.3, 130.6 and 134.0 ppm in the resin and 100.0 and 134.0 ppm in the asphaltene, likely represent aniline adducts of a specific constituent that occurs at relatively high concentration in the fractions. The presence of the anilinohydroquinone peak in the resin provides evidence for the occurrence of quinones in this fraction. Interestingly, the resin shows a greater proportion of heterocyclic nitrogens than the asphaltene. This may possibly be explained by the greater aliphatic carbon content, and by implication, greater activated methylene carbon content of the resin compared to the asphaltene. The configurations of carbonyl groups that condense with aniline to form indoles and quinolones in the Bischler and CLK reactions contain activated methylene carbons (Fig 12).

Similar to the case with hydroxylamine, the increase in elemental nitrogen contents on derivatization with aniline are low. For both the asphaltene and resin, the nitrogen contents increase by only a tenth of a percent (Table 1).

## Discussion

The ^15^N NMR analyses have confirmed the presence of ketones and provided strong evidence for the presence of quinones in the asphaltene and resin samples. The presence of hydroxamic acid peaks in the spectra of the hydroxylamine reacted samples is evidence for the occurrence of esters in the asphaltene and resin. Previous approaches to carbonyl analysis in asphaltenes have included derivatization with hydroxylamine followed by methylation, or derivatization with methoxylamine, in conjunction with ^1^H or ^13^C NMR detection of the = N-OCH_3_ groups [30]. The ability to detect reactions of hydroxylamine with carbonyls other than ketones, such as esters, and to detect the secondary reactions of the oximes, is an advantage to following the ^15^N label. Speight noted the limited information on the location of oxygen functionalities in asphaltenes [4]. Some possible configurations of the ketone groups in the asphaltene and resin fractions can be inferred from a consideration of the likely reactions that lead to heterocyclic condensation products with aniline and to the Beckmann reaction products with hydroxylamine. These include aromatic ketones and ketones adjacent to quaternary carbon centers such as β-hydroxyketones, β-diketones, and β-ketoesters.

The carbonyl groups in the asphaltene and resin fractions susceptible to nucleophilic addition and substitution reactions by hydroxylamine and aniline also constitute potential substrate sites for condensation with ammonia. Where ammonia based fertilizers are applied to enhance biodegradation of crude oil contaminating soils, sediments and natural waters, there is the possibility for ammonia fixation by the resins and asphaltenes.

The ^15^N NMR approach is amenable to further development in several respects. An intensity standard for spin-counting experiments in the solid-state could be used to quantitate the incorporation of hydroxylamine and aniline into the samples as a comparison to the elemental analysis data. The oximated resin sample was adsorbed to alumina for solid-state NMR analysis in this study. Use of a sealable pyrex insert for the solid state rotor or analysis by HRMAS (high resolution magic angle spinning) ^15^N NMR spectroscopy are potential alternatives to the solid phase support. Further refinement of the liquid-state ^15^N NMR analyses would include use of higher fields and indirect detection techniques. There is potential for combining FTICR-MS with the ^15^N NMR analyses to determine the percentage of the asphaltene and resin molecules that react with the hydroxylamine and aniline, and the number of carbonyl groups per molecule that are derivatized.

## Abbreviations

NMR: Nuclear Magnetic Resonance
CP/MAS: cross polarization/magic angle spinning
FTICR-MS: Fourier transform ion cyclotron resonance mass spectrometry
AFM: atomic force microscopy
DEPT: distortionless enhancement by polarization transfer
